# The rate of estrogen receptor‐conversion associated with tumor progression in estrogen receptor‐positive breast cancer patients following adjuvant Tamoxifen administration

**DOI:** 10.1002/cnr2.1431

**Published:** 2021-06-04

**Authors:** Sirus Djahansouzi, Bettina Hanstein, Daniel Rein, Michel Clees, Werner Rath

**Affiliations:** ^1^ Centre Hospitalier Emile Mayrisch, Department of Obstetrics & Gynecology Rue Emile Mayrisch Esch‐sur‐Alzette Luxembourg; ^2^ Department of Obstetrics & Gynecology University Hospital Cologne Köln Germany; ^3^ Department of Obstetrics & Gynecology University Hospital Düsseldorf Düsseldorf Germany; ^4^ Department of Obstetrics & Gynecology University Hospital Aachen Aachen Germany

**Keywords:** biomarkers, breast cancer, estrogen receptor, progesterone receptor, receptor conversion, Tamoxifen

## Abstract

**Background:**

Hormone Receptor (HR)‐discordance between primary breast cancer and metastasis is a known biological phenomenon. Discordance studies usually comprise a heterogeneous group of HR‐positive and negative patients and allow for the comparison of changes in HR‐status from the primary to the recurrent disease. However, in a clinical setting, the rate of estrogen receptor‐conversion following endocrine therapy with agents such as Tamoxifen (TAM) in estrogen receptor‐positive cancers is of primary interest as opposed to total receptor discordance.

**Aim:**

To investigate the rate of estrogen receptor‐conversion associated with tumor progression in estrogen receptor‐positive breast cancer patients following adjuvant TAM administration and to compare the results with the meta‐analysis data of HR‐discordance studies.

**Methods and Results:**

A retrospective *double‐center* review of biomarkers in 67 estrogen receptor‐positive breast cancer patients who underwent TAM treatment in the adjuvant setting. The estrogen and progesterone receptor‐status were compared at the time of diagnosis and following relapse and the Disease‐free Survival, mean duration of TAM treatment as well as the operative, radiation, and cytotoxic therapies registered before TAM treatment, were recorded.

Initially, all patients were estrogen receptor‐positive. The average age at the time of diagnosis was 52.8 ± 12.4 years. After recurrence, only 47 patients (70.1%) were still estrogen receptor‐positive with a highly significant loss of estrogen receptor‐expression in 29.9% of cases. The mean duration of TAM treatment was 40.7 ± 19.9 months. 45 patients (i.e., 67.2%) progressed during the TAM treatment and the remaining 22 patients (32.8%) developed relapse after the TAM treatment had finished. Initially, there were 82.1% progesterone receptor‐positive and 17.9% progesterone receptor‐negative, but after relapse the progesterone receptor‐positive cases diminished significantly to 53.7%, showing a progesterone receptor‐loss of 28.4%.

**Conclusion:**

The rate of estrogen receptor‐loss associated with tumor progression following TAM treatment is approximately 30%, which is of clinical relevance in order to evaluate further endocrine efficacy in these patients. This rate of receptor conversion is roughly 6‐13% higher compared to the recently published meta‐analysis data of discordance studies. This discrepancy could possibly be due to anti‐hormonal therapy with TAM accentuating receptor conversion.

## 1 INTRODUCTION

Estrogen (E) or Progesterone (P) are a family of steroid hormones that serve as a stimulus for tissue growth and differentiation in the breast and many other tissues. Likewise, in breast cancer tissue, these hormones stimulate the growth of malignant cells.^1^ The blockade of the Hormone Receptor (HR) activity in cancer cells with anti‐hormonal therapy (AHT), such as Tamoxifen (TAM), presents an effective clinical treatment. TAM is a Selective Estrogen‐Receptor Modulator (SERM) that binds to the intracellular Estrogen Receptor (ER) and acts as a competitive antagonist by binding to the Ligand‐Binding Domain (LBD) and promoting ER conformational changes, thereby inhibiting co‐activator‐binding to the receptor. This hinders the propagation of downstream signaling pathways and gene expressions coupled with ER, which will lead to cell growth and division.^2^, ^3^

TAM is the first‐line agent of choice for the treatment of HR‐positive breast cancer, especially in pre‐ and perimenopausal women. The Early Breast Cancer Trialists Collaborative Group^4^ have demonstrated a 50% reduction in breast cancer mortality in a meta‐analysis of 15 years follow‐up data from randomized trials of patients with ER‐positive disease, given 5 years adjuvant TAM after 6 months of anthracycline‐based chemotherapy.

ER and/or Progesterone Receptor (PR)‐status are important biomarkers in breast cancers and, at present, defined clinically as the presence of at least 1% positive staining in tumor nuclei through Immuno‐Histo‐Chemistry (IHC) testing. This is currently the cut‐off for HR‐positive cancers in the clinical setting and is in accordance with major guidelines such as ASCO, ESMO, German AGO, and the St. Gallen International expert consensus for the use of endocrine therapy in these patients.^5^, ^6^ Formerly, the cut‐off limit was set at 10% positive cells in IHC, which from a study point of view is much better for distinguishing between real positive HR‐status and borderline cases. Nonetheless, the ER‐expression serves as a predictive and prognostic biomarker and ER‐positivity has been found to confer a survival advantage in patients treated with endocrine therapy, specifically TAM, which has not been observed in the ER‐negative disease.^7^

Microarray studies differentiate gene expression patterns of cells and have helped to distinguish breast cancer into various intrinsic subtypes. These different subtypes are referred to as Luminal A, Luminal B, HER2‐enriched, Triple‐negative/basal‐like, or normal‐like. Some gene expression subtypes that are referred to as Luminal A, Luminal B, normal‐like, code for HR‐positive breast cancers and present a major treatment option in these patients for AHT.^8^, ^9^

There are genetic events that alter the generation of estrogen and the metabolism of TAM in breast cancer patients. A recent report indicated that the aromatase gene responsible for the generation of estradiol in postmenopausal women, called CYP19A1, may be amplified in endocrine‐resistant breast cancer.^10^ Another known phenomenon is the metabolism of TAM to a more active metabolite, called Endoxifen, via cytochrome P450 2D6 (CYP2D6).^11^ Evidence suggests that polymorphism, existing for the CYP2D6‐gene, can lead to either a reduced or increased metabolism to the active metabolite Endoxifen and hence show better or worse clinical outcomes in these cancer patients.

Although there has been enormous progress in the treatment of HR‐positive breast cancer, the resistance to drug treatment remains a problem. The Early Breast Cancer Trialists Collaborative Group^12^ recently reported a meta‐analysis based on 20 years follow‐up of 88 clinical trials that involved 62 923 women with ER‐positive breast cancer treated for 5 years with AHT, mainly TAM. Breast cancer recurrences occurred at a steady rate throughout the period of 5‐20 years. There are several mechanisms that HR‐positive breast cancers may adapt to resist treatment. The most common mechanism for the lack of efficacy of AHT seems to be the loss of the HR‐expression, dysregulation of co‐regulators, and cross talk with growth factor signaling pathways.^2^, ^13^ Furthermore, the loss of the ER from the adjuvant to the metastatic setting seems to be associated with worse overall survival and therefore the ER‐status seems to be a predictor of survival, whereas changes to the PR from the adjuvant to the metastatic setting were not associated with a change in survival.^14^

There are several studies that have shown the ER‐evolution in HR‐positive breast cancer patients. The retrospective study by Broom et al^15^ comprised the data of 100 patients with breast cancer, of which 73 were initially ER‐positive and were consequently followed up. After progression, they observed a change in ER‐status in 17.7% of cases (switching occurred both from ER + →ER‐ and vice versa) and a reduction of 37.3% for PR (all tumors lost the PR). The problem with the study was that the authors did not elaborate precisely on the adjuvant treatment that each patient received, presumably AHT for the ER‐positive patients, and since the cohort chosen was heterogenic in terms of ER‐status, there were also some patients with increased ER‐expression after relapse. Hull et al^16^ published their results of 232 patients, where they found a discordance rate of 19% for patients losing ER and 13% for those gaining ER with time. By contrast, Lower et al^14^ found a discordance rate for ER of 30% in their review of 200 patients with 19.5% of tumors losing ER and 10.5% gaining ER. For PR, the researchers found a discordance rate of 39.3%. A meta‐analysis by Franco et al^17^ totaling 658 paired ER samples and 418 PR samples found a discordance rate of 29% and 27% for ER and PR respectively. More recent data from a study by Lindström et al^18^ encompassing 459 patients showed a conversion rate from primary ER‐positive to relapse ER‐negative as being at 24.6% and ER‐positive staying as ER‐positive at 47.1%, while finding that the conversion rate from primary ER‐negative to relapse ER‐positive was 7.8% and that of primary ER‐negative staying ER‐negative was 20.5%. It should be noted, that none of the above studies elaborated exactly on the administration and duration of the AHT in their collectives.

In many of these studies, the common denominator was always the presence of HR‐negative patients in their initial collective, some of which turned from ER‐ or PR‐negative to positive over time. As one has to assume that the HR‐negative patients did not receive AHT, a switch from negative to positive may be better explained by the tumor biology of progression, rather than the specific clinical effects that one might observe due to adjuvant therapy.

Furthermore, clinically the total rate of discordance is of limited value, since it measures changes to ER‐status from ER‐positive to ER‐negative and vice versa as well as changes from PR‐positive to PR‐negative and vice versa. For the clinician, it is more pragmatic to know the rate of ER‐loss that is, ER + →ER‐, following adjuvant AHT with endocrine agents such as TAM, as this information is pertinent for the use of endocrine therapy in relapsed patients.

The aim of the study was to investigate the rate of ER conversion in ER‐positive breast cancer patients from the adjuvant to recurrent disease, when subjected to TAM therapy, as one would find in a clinical setting. At the same time, any concomitant changes that might occur to PR‐status in this patient cohort were monitored. Finally, the data were compared with the published meta‐analysis of discordance studies from 2018.

## 2 PATIENTS AND METHODS

### 2.1 Data source

The study was a retrospective *double‐center* study and the acquisition of patient data was between May 2000 and September 2008 from the archives of the University Hospital in Düsseldorf and University Hospital of Aachen in Germany. For analysis purposes, the data of the centers were pooled together. Prior to conducting the analysis, research ethics board approval from both the institutions involved was obtained. Patient consent was waived in order to review their medical data in this retrospective study. Patient confidentiality was respected throughout the investigation.

### 2.2 Selection of cases

**FIGURE 1:**
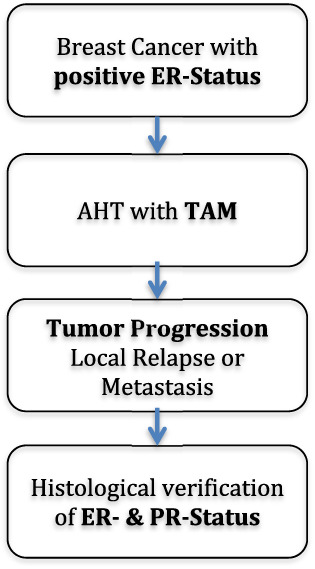
Flowchart showing the algorithm and necessary criteria for inclusion in the study

The search criteria entailed identification of patients with primary ER‐positive breast cancers, with a complete file and documentation, including adequate histology, PR‐status, age, and co‐morbidity who underwent TAM treatment in an adjuvant setting and progressed under or after treatment with TAM. All of the patients were again identified with histological sampling and identification of the ER‐ and PR‐status in recurrent disease. A total of 67 patients, which fully matched the inclusion criteria and had a complete and well‐documented file, were selected. The distribution of patient recruitment was as follows: 68.7% from the archives of the University Hospital of Düsseldorf and 31.3% from the archives of the University Hospital of Aachen. The algorithm and inclusion criteria needed to qualify for the study are listed in Figure 1.

The patients that were found to qualify for the cohort study were then reviewed and the required demographic information was extracted from their medical records as listed in Table 1.

**TABLE 1:** Demographic information collected for each patient that entered the study

Parameters investigated
Gender
Date of primary diagnosis
Age at primary diagnosis
Menopausal status
Localization of the cancer
Histology of the primary tumor and of the relapse
Grading of the tumor before and after relapse
Initial stage of disease / TNM‐classification
ER‐ & PR‐status at the beginning and after relapse
Type of primary surgery
Neoadjuvant or adjuvant Chemotherapy
Adjuvant radiation therapy
The duration of AHT with Tamoxifen
Major Co‐morbidities
Disease‐free Survival
Locale relapse or metastasis
Site of metastasis: bone, brain, liver, lung, other
Time elapsed between the end of AHT with TAM and relapse

All patients selected had breast cancer stage I‐III with a complete TNM classification (AJCC/UICC). All patients underwent an R0 resection of primary cancer. The data does not specify the type of lymph node resection carried out. Patients that had documented interruptions in their AHT with TAM were excluded from the study.

### 2.3 Histopathological tumor grading and HR‐status

Histopathological grading classification of tumors (according to Elston/Nottingham modification of Scarff–Bloom–Richardson)^19^ and ER‐ and PR‐analysis were performed in the Pathology Departments of the respective University Hospitals at the time of presentation of the primary or recurrent disease. The assay used for the analysis of the HR‐status was the IHC assay as described elsewhere.^20^, ^21^ For the analysis of the ER‐ and PR‐expression in breast cancer cells, the color intensity of the cell stains and the percentage of stain‐positive cells have been multiplied to give the so‐called Immunoreactive (IRS)‐Score, as shown in Table 2.

**TABLE 2:** IRS‐score reporting a point system for staining of HR‐positive cells

Points	Color intensity of cells	Percentage (%) positive cells
0	No staining	0
1	Weak	≤10
2	Moderate	11‐50
3	Strong	51‐80
4	‐	81‐100

For the evaluation of the ER‐ and PR‐status in our collective of breast cancer patients, the tumor was classified as HR‐positive, if the associated IRS‐score was ≥3 and at least 10% of the tumor cells stained positive for the HR. Scores of ≤3 and HR‐expression less than 10% were regarded as negative.

Because the reporting of HR‐status has changed over time, the HR interpretation was considered positive or negative based on the standard criteria at the time of the evaluation (2008). Therefore, the cut‐off for ER‐positivity was chosen at 10%, since it adhered to the international guidelines asserting that 10% or higher staining of cancer cells can be interpreted as a real positive ER‐status. The low staining ER‐positive tumors with a lower ER‐expression between 1%‐10% were not considered as adequate to be included in the study.

### 2.4 Statistical analysis

Wherever appropriate, the data in this study is quoted as the mean with its corresponding Standard Deviation. The statistical analysis was carried out using the non‐parametric test of McNemar applied to paired frequencies.^22^ Statistical significance is assumed for *p* ≤ .05.

## 3 RESULTS

### 3.1 Study characteristics

A total of 67 patients were identified and analyzed. These patients had received 20 mg TAM per day as standard adjuvant treatment for ER‐positive breast cancer.

The study group was comprised of 66 females (98.5%) and 1 male patient (1.5%). The average age at the time of diagnosis was 52.8 ± 12.4 years. The histology was comprised of 43.3% ductal, 13.4% lobular, 7.5% others, and 35.8% as unidentifiable from the files.

There were 65 patients (97%) in the primary situation and 2 patients with local relapse (3%) receiving TAM. At the time of the primary diagnosis, 22.4% were at stage I, 50.7% at stage II, 13.4% at stage III. In 13.4% of the patients, the stage of the disease was not evaluable. All patients underwent surgery, in which 53.7% were treated by breast‐conversing surgery and 44.8% had a mastectomy. An axillary dissection / sentinel node biopsy was carried out in 89.6% of the patients. 59.7% of patients received adjuvant chemotherapy and 56.7% of patients had radiation therapy before commencing the treatment with TAM. The chemotherapy protocols varied and comprised the substances Epirubicin, Docetaxel, Paclitaxel, 5‐FU, Methotrexate, Cyclophosphamide, and /or Trastuzumab.

The mean Disease‐free Survival (DFS) was 54.9 ± 34.6 months. All the demographic data are summarized in Table 3.

**TABLE 3:** Demographic data of the patients investigated in the study

Feature	Value
Patients studied (*n*)	67
Gender	66 female, 1 male
Age at primary diagnosis (years)	52.8 ± 12.4
DFS (month)	54.9 ± 34.6
Tumor Stage at diagnosis (% of patients)	
I	22.4
II	50.7
III	13.4
Unidentifiable	13.4
Histology of breast cancers (% of patients)	
Ductal	43.3
Lobular	13.4
Others	7.5
Unidentifiable	35.8
Primary surgery (% of patients)	100
Breast conserving	55.2
Ablative	44.8
Axillary dissection/Sentinel‐biopsy	89.6
Chemotherapy (% of patients)	59.7
Adjuvant	49.3
Neoadjuvant	10.4
Radiation therapy (% of patients)	56.7

### 3.2 The duration of TAM treatment and sites of metastasis

The mean duration of TAM administration was 40.7 ± 19.9 months. For the patients that relapsed after finishing the TAM treatment, the duration of TAM administration was a maximum of 5 years, which adhered to the standard guidelines and recommendations for TAM treatment at the time of the evaluation.

A total of 45 patients (i.e., 67.2%) progressed during the AHT with TAM. This subset consisted of 34 patients (50.7%) having a local recurrence and 11 patients (16.4%) with distant metastasis. By contrast, 22 patients (32.8%) developed a relapse after the TAM treatment had finished. This subset consisted of 15 local recurrences (22.4%) and 7 patients with distant metastasis (10.4%). The timeline for tumor progression is depicted in Table 4.

**TABLE 4:** The distribution of the frequencies of the breast cancer relapses

Patients relapsed under Tam (%)	Patients relapsed after Tam (%)
Total 67.2	Total 32.8
Local relapse 50.7	Local relapse 22.4
Metastasis 16.4	Metastasis 10.4

A total of 49 patients (73.2%) had a local relapse, while 18 patients (26.8%) developed the metastatic disease at the time of progression and the sites of metastatic tumor expressed as a percentage of total number of patients were: bones (9%), liver (4.5%), brain (1.5%), and others (12%). This distribution is shown in Table 5.

**TABLE 5:** The distribution of the sites of metastasis after tumor progression

Site of metastasis	% of total patients
Bone	9
Liver	4.5
Brain	1.5
Others	12

### 3.3 The distribution of histopathological grading from the primary to relapse setting

Analyzing the tumor patients with regards to histopathological grading before treatment, the following distribution was obtained: 4.5% were grade I, 3% were grade I‐II, 37.3% were grade II, 1.5% were grade II‐III, 25% were grade III and there were 28.4% of patients with non‐evaluable grading. With recurrent disease, the grading changed, showing no patient with grade I, 20.9% with grade II, 4.5% with grade III, and 74.6% of patients with unknown or undocumented grading status. The distribution of grading in the cancers is shown in Table 6.

**TABLE 6:** The distribution of grading in breast cancers before and after AHT with TAM

Grading	Primary tumor (% of patients)	Relapse (% of patients)
I	4.5	0
I‐II	3	0
II	37.3	20.9
II‐III	1.5	0
III	25.0	4.5
unidentifiable	28.4	74.6

Out of the 17 patients whose tumors had documented grading before and after TAM, there were 10 patients whose grade of differentiation did not alter. From the remaining 7 cases, a change in the grading was noted. In 3 out 7 patients the grading increased and in 4 cases the grading decreased, that is, the differentiation became better. Out of the 3 patients whose grade of differentiation became worse, in 2 cases a change in the HR‐status was also observed while in 1 case HR‐status remained unaltered. Out of the 4 patients whose grading decreased, 3 had no change in the HR‐status and in only 1 case had the HR‐status changed.

### 3.4 ER‐ and PR‐loss from primary breast tumor to recurrent disease

**FIGURE 2:**
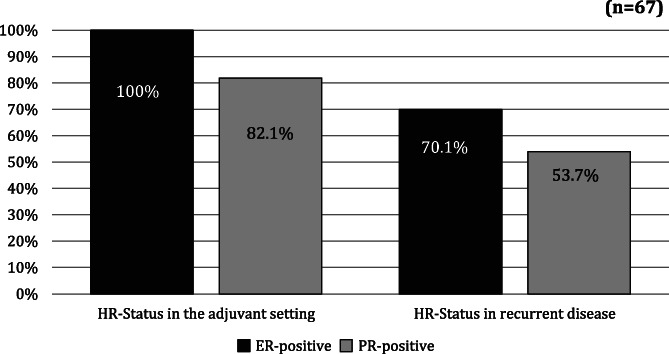
The rate of ER‐ und PR‐conversion from the primary to recurrent disease, in patients administered adjuvant TAM therapy [Correction added on 10 September 2021, after first online publication: Figure 2 was changed to black and white.]

Initially, all patients were ER‐positive, but with the recurrent disease only 47 patients (70.1%) were still ER‐positive. In 20 patients (i.e., 29.9%) the ER‐status changed from positive to negative and this change was statistically highly significant (*p* ≤ .001). At the start of the study, there were 55 patients with positive PR‐status (82.1%) and 12 patients with negative PR‐status (17.9%). After relapse the positive PR‐status diminished significantly to 36 cases (53.7%), resulting in a loss of 28.4% while the PR‐negative cases increased to 31 cases (46.3%). Applying the McNemar test, a χ^2^ = 19.0125 (degrees of freedom = 1) was computed for the ER‐group and a χ^2^ = 18.1071 (degrees of freedom = 1) for the PR‐group. This resulted in a probability of *p* < .001 in both groups, thus confirming a highly significant reduction in both ER‐ und PR‐status from the adjuvant to recurrent disease, in patients administered TAM treatment. This change is depicted in Figure 2.

## 4 DISCUSSION

Progression of disease or recurrence usually indicates either a selection of clones that are resistant to treatment or evolution of the primary tumor with accumulation of mutations that subsequently lead to therapy resistance. One can also further subdivide resistance into intrinsic resistance, which is present directly after initiation of therapy, while acquired resistance develops after initial response. Examples of acquired resistance include breast cancer evolution in response to various pressures, such as the lack of nutrients or oxygen as well as in response to treatment with targeted therapies.^23^ Part of this evolution may also be explained by the loss in expression of ER and PR in tumor cells and splicing variants as well as altered sensitivity and activation of proliferating pathways (EGFR, PI3K/AKT/mTOR, RAS/Raf/MAPK).^2^

There are multiple other mechanisms of breast cancer resistance to AHT and clinical genomic testing of cancers may become very important. Mutations in metastatic breast cancer may also include mutations in the gene of Estrogen‐Receptor 1 (ESR1),^24^ some of which confer a shortened Overall Survival (OS). Other mutations can be present in the LBD and cause ER to exhibit Ligand‐independent activity. Reports of recurrent ESR1‐gene fusions, whereby the LBD of the receptor is removed, can result in a constitutively active ER in endocrine‐resistant breast cancer.^25^ ESR1 mutations have also been demonstrated in patients with ER‐positive disease previously exposed to Aromatase Inhibitor (AI) therapy and found to confer a shorter progression‐free survival with subsequent AI therapy.^23^, ^26^

The latest and largest meta‐analysis to date, published in 2018 by Schrijver et al,^27^ *pooled* the data from 39 different studies encompassing 1948 patients for ER‐analysis from 1986‐2016. They showed that the total discordance percentage for ERα (i.e., from the primary to distant metastasis) varied between different studies from 7.3% to 51.2%, with a *pooled* random effect (conservative random effect model) percentage of 19.3% (95% Cl = 15.8% to 23.4%). The percentage conversion from ER + →ER‐ was 22.5% (95% Cl = 16.4% to 30.0%) and from ER‐ → ER+ was 21.5% (95% Cl = 18.1% to 25.5%). They further divided studies into two groups, one group for which the threshold of ER‐positivity was set at 1% in the IHC, and another group, for which the ER‐positivity was set at 10% in the IHC. They showed a *total pooled* ERα‐conversion percentage (ER + →ER‐) of 17.7% (95% Cl = 13.5% to 22.7%) for the 1% cut‐off in IHC and 19.4% (95% Cl = 11.5% to 24.2%) for the 10% cut‐off in IHC. However, these groups showed no significant difference statistically. Moreover, the frequency of conversion in the subgroup analysis ER + →ER‐ was given as 16.9% (95% Cl = 11.5% to 24.2%) for the 1% threshold of ER‐positivity and 23.9% (95% Cl = 15.7% to 34.7%) for the 10% threshold of ER‐positivity. Conversion from ER‐ → ER+ occurred in 22.6% and 17.3% of tumors for the respective subgroups (i.e., 95% Cl = 17.9% to 28.0% for the 1% threshold; 95% Cl = 11.7% to 24.8% for the 10% threshold). Finally, they showed that ERα‐discordance was statistically significantly higher in the central nervous system and bone compared with liver metastasis. Unfortunately, there were no exact data on the extent and specific use of AHT in the subgroup of patients with ER‐positive disease.

Similarly, the researchers analyzed 1730 patients for PR, showing an overall conversion rate from the primary to the metastatic setting as being 30.9% (95% Cl = 26.6% to 35.6%). Their data analysis is also very much in line with the findings of PR‐loss in this study. The demographic data of the meta‐analysis in terms of mean age at diagnosis of the primary tumor, the histological type of cancers and the DFS is in accordance with our data.

However, most discordance studies initially include all breast cancer patients in their studies (HR‐positive as well as HR‐negative), monitor the conversion to the HR over time, and make a sum of the conversion rates for ER and PR to attain a discordance rate, whereas the biology of the cancers and their treatment may vary considerably between the patients depending on the HR‐status. Therefore, these groups are very heterogeneous with regards to their therapy modalities, such as AHT, since patients with HR‐negative breast cancers have also been included. Consequently, while one can certainly conclude that there is a significant change in the HR‐status in these heterogeneous groups over time, this HR evolution is partly a reflection of the tumor biology over time, as opposed to influences of adjuvant therapies.

Several other reports demonstrate an altered HR‐status throughout tumor progression, which significantly and negatively affects survival. The study by Stueber et al^28^ reports HR‐change (sum of ER‐ and PR‐discordance) at 33%, Broom et al^15^ show a discordance rate of 17.7% (tumors changing from ER + →ER‐ and vice versa) and Lower et al^14^, ^29^ reports ER‐discordance between primary and metastasis at 30%. The large cohort study by Lindström et al^18^ monitored several subgroups of receptor discordance, whereby the most clinically relevant subgroup of patients, that is, ER + →ER‐, which received endocrine therapy from the primary to recurrent disease, had a conversion rate of 24.6%. They also used a 10% cut‐off for ER‐positivity in their definition of positive ER‐cancers. The researchers were further able to gather data on the influence of adjuvant therapy with regards to change in the ER‐status. They showed that ER‐loss was at 34.3% in patients receiving AHT and chemotherapy, 29.0% in patients receiving solely AHT, 19.8% in patients receiving solely chemotherapy, and only 11.5% in patients receiving no adjuvant treatment (*n* = 10), thereby inferring both biologic and therapeutic influences on ER‐conversion.

The recent study by Stueber et al^28^ in 2019 had a similar approach to ours, since the researchers carried‐out a retrospective analysis of the relapses in HR‐positive breast cancer patients. The study design included ER‐positive *and*/*or* PR‐positive patients and the threshold for HR‐positivity was set at ≥1%, which is currently the definition for HR‐positive breast cancer. They monitored 165 patients who received some form of AHT, 57 patients received TAM, 35 received AI and 71 patients received several different adjuvant endocrine therapies. They demonstrated a pooled discordance rate of 29.1% in ER‐, PR‐, or HER2‐status. The patients were subjected to an array of different AHT such as TAM, AI, TAM + AI, TAM + GnRH, AI+GnRH, which could introduce some inaccuracy in comparison to the setting of this study. Since the authors used the current clinical definition of HR‐positive breast cancer as the basis of their study, this meant that they included some patients who were PR‐positive and ER‐negative.

The sum of receptor discordance that is, the addition of conversion of the ER‐status from ER‐positive to ER‐negative and vice versa as well as changes from PR‐positive to PR‐negative and vice versa are clinically of limited value, due to the heterogeneity of the cancers involved. The inclusion of progesterone discordance and the reporting of a total discordance rates is also difficult to interpret, since changes to progesterone receptor‐status do not correlate with a change in survival.^12^ Furthermore, the change of ER‐ or PR‐negative disease to positive is also clinically unclear, since HR‐negative disease such as Triple‐negative and HER2neu cancers have other tumor biology and are not candidates for endocrine therapy.

In a clinical setting, it is more advantageous to monitor the conversion of ER‐positive cancers that is, Luminal A and B tumors, since they are the primary targets for AHT and herewith show better survival data.^4^, ^18^, ^29^ This study has included only ER‐positive patients who received 20 mg TAM/day in the analysis. ER‐positivity was defined as a threshold of ≥10% positive cells in IHC. In this homogenous setting, the ER‐loss was recorded at 29.9%. This finding is of practical relevance for clinicians, since it helps to estimate the effectiveness of future AHT in initially ER‐positive disease, especially in cases where biopsy is not readily available. Our data is comparable with the data of Lindström et al^18^ in terms of change from ER + →ER‐ in the subgroup of patients receiving endocrine therapy. One difference to this study is that Lindström et al^18^ did not elaborate exactly on the extent and the use of different endocrine substances, but this study shows the rate of ER‐loss associated with TAM alone.

The value of ER‐loss (29.9%) in this study needs further interpretation and comparison to the results of the meta‐analysis data by Schrijver et al.^27^ Setting aside total receptor discordance and analyzing the relevant subgroup of patients where ER + →ER‐, the authors observed the following statistic: conversion from ER‐positive to negative as 16.9% for the 1% threshold of ER‐positivity and 23.9% for the 10% threshold of ER‐positivity.

It must be noted that the population of patients in the meta‐analysis was very heterogeneous and it incorporated the data of 39 studies, some newer and some older, and there was no exact definition of the adjuvant therapy in terms of AHT the patients received in this subgroup. Therefore, at best, only a portion of patients received AHT and hence the data cannot be directly compared to that in this study. Interestingly, the meta‐analysis data showed that in the subgroup with 10% as a threshold for ER‐positivity, the ER conversion rate was markedly higher than that for 1% ER‐positivity threshold, and the value becomes closer to the estimates in this study.

Although there are limitations to this study due to the small number of cases and retrospective design, this study primarily used only a homogenous ER‐positive cohort, fixed the variable of treatment to TAM, and used a high threshold of 10% as criteria for ER‐positivity. This setting allows for differences to classic discordance studies to be accentuated. As in any experimental setting, it is fundamentally important to compare a treatment arm with a control arm to accentuate differences. Since a control arm with ER‐positive patients devoid of TAM administration is unethical, the best replacement at present would be a comparison to the meta‐analysis data.

We are bringing forward this data at the present time, in order to compare our data in corroboration with the recent large‐scale meta‐analysis. With the above‐mentioned limitations, the discrepancy between ER‐loss in this study and the meta‐analysis when adjusted for ER‐loss that is, ER + →ER‐, is approximately between 6%‐13%. This discrepancy may be explained by several possibilities. Assuming a flawless meta‐analysis, then the discrepancy could be either due to methodological shortcomings in this study or that TAM acts as a stress‐factor and maybe a significant driving force for breast cancer cells to further lower their ER‐expression in order to accommodate this pharmacological stress. Interestingly, Lindström et al^18^ showed, in their retrospective analysis of 459 ER‐positive patients, that AHT was associated with a significant higher proportion of patients losing ER.

This present study concludes that as a rule of thumb, TAM administration in the adjuvant setting, results in a 30% loss of Estrogen‐receptor expression in ER‐positive patients following tumor progression and this information is pertinent for a clinician to judge the further use of endocrine therapy in metastatic and recurrent breast cancers, where biopsy is not readily available.

These differences warrant further and larger investigations such as prospective randomized trials and molecular biology testing to elaborate as to what extent different AHT may influence ER‐loss beyond tumor biology or evolution.

## CONFLICT OF INTEREST

The authors have no disclosures and declare to have no conflicts of interests in connection with this article.

## AUTHOR CONTRIBUTIONS

All authors had full access to the data in the study and take responsibility for the integrity of their data and the accuracy of the data analysis. *Conceptualization*, S.D., B.H. and D.R.; *Methodology*, S.D., B.H., and D.R.; *Investigation*, S.D., B.H., D.R., W.R., and M.C.; *Resources*, S.D., D.R., B.H., and W.R.; *Writing‐original draft*, S.D., M.C., and W.R.; *Writing‐review & editing*, S.D., M.C.; *Visualization*, S.D. and M.C.; *Supervision*, W.R. and D.R.

## ETHICS STATEMENT

The approval for this study was obtained from the Research Ethics Board of the University of Düsseldorf and the University Hospital of Aachen. The need for patient consent was waived, given the deidentified nature of the data to be retrospectively collected about patients' routine clinical care.

## Data Availability

The data that supports the findings of this study is available from the corresponding author upon reasonable request.
